# The efficacy of prospective memory rehabilitation plus metacognitive skills training for adults with traumatic brain injury: study protocol for a randomized controlled trial

**DOI:** 10.1186/s13063-016-1758-6

**Published:** 2017-01-05

**Authors:** Jennifer Fleming, Tamara Ownsworth, Emmah Doig, Lauren Hutton, Janelle Griffin, Melissa Kendall, David H. K. Shum

**Affiliations:** 1 School of Health and Rehabilitation Sciences, University of Queensland, Brisbane, 4072 QLD Australia; 2 Menzies Health Institute Queensland and School of Applied Psychology, Griffith Health Institute, Griffith University, Mount Gravatt, QLD Australia; 3 Occupational Therapy Department, Princess Alexandra Hospital, Brisbane, Australia; 4 Acquired Brain Injury Outreach Service, Princess Alexandra Hospital, Metro South Hospital and Health Service, Woolloongabba, QLD Australia; 5 Menzies Health Institute Queensland and School of Human Services and Social Work, Griffith University, Meadowbrook, QLD Australia

**Keywords:** Brain injury, Prospective memory, Self-awareness, Rehabilitation, Randomized controlled trial

## Abstract

**Background:**

Impairment of prospective memory (PM) is common following traumatic brain injury (TBI) and negatively impacts on independent living. Compensatory approaches to PM rehabilitation have been found to minimize the impact of PM impairment in adults with TBI; however, poor self-awareness after TBI poses a major barrier to the generalization of compensatory strategies in daily life. Metacognitive skills training (MST) is a cognitive rehabilitation approach that aims to facilitate the development of self-awareness in adults with TBI. This paper describes the protocol of a study that aims to evaluate the efficacy of a MST approach to compensatory PM rehabilitation for improving everyday PM performance and psychosocial outcomes after TBI.

**Methods/design:**

This randomized controlled trial has three treatment groups: compensatory training plus metacognitive skills training (COMP-MST), compensatory training only (COMP), and waitlist control. Participants in the COMP-MST and COMP groups will complete a 6-week intervention consisting of six 2-h weekly training sessions. Each 1.5-h session will involve compensatory strategy training and 0.5 h will incorporate either MST (COMP-MST group) or filler activity as an active control (COMP group). Participants in the waitlist group receive care as usual for 6 weeks, followed by the COMP-MST intervention. Based on the sample size estimate, 90 participants with moderate to severe TBI will be randomized into the three groups using a stratified sampling approach. The primary outcomes include measures of PM performance in everyday life and level of psychosocial reintegration. Secondary outcomes include measures of PM function on psychometric testing, strategy use, self-awareness, and level of support needs following TBI. Blinded assessments will be conducted pre and post intervention, and at 3-month and 6-month follow-ups.

**Discussion:**

This study seeks to determine the efficacy of COMP-MST for improving and maintaining everyday PM performance and level of psychosocial integration in adults with moderate to severe TBI. The findings will advance theoretical understanding of the role of self-awareness in compensatory PM rehabilitation and skills generalization. COMP-MST has the potential to reduce the cost of rehabilitation and lifestyle support following TBI because the intervention could enhance generalization success and lifelong application of PM compensatory strategies.

**Trial registration:**

New Zealand Clinical Trials Registry, ACTRN12615000996561. Registered on 23 September 2015; retrospectively registered 2 months after commencement.

**Electronic supplementary material:**

The online version of this article (doi:10.1186/s13063-016-1758-6) contains supplementary material, which is available to authorized users.

## Background

Memory problems are the most common cognitive complaint in people with traumatic brain injury (TBI) [1, 2]. Prospective memory (PM) refers to the ability to remember to carry out a planned action or intention at a future point in time [3]. Examples of PM are remembering to pay a bill by the due date, remembering to take medication each morning, or remembering to pass on a message the next time you see a particular person. Successfully remembering to perform an intended action is a critical cognitive skill in daily life. We are constantly making mental notes of tasks that we need to complete in the future, and failures of PM are associated with feelings of having let oneself or others down. Despite its relevance to daily life, PM is a relatively new construct in the scientific literature compared to retrospective memory (RM), which involves the recall or recognition of previously learnt information and past events. However, over the past 10 years research on PM has grown rapidly. Research has shown that people with TBI experience debilitating PM deficits [4–6] which impact on independent living (e.g., requiring a carer at home), social participation (e.g., failure to keep engagements), and employability (e.g., workers are seen as unreliable) [7]. Therefore, PM impairment is a target for rehabilitation efforts which often focus on the use of compensatory strategies to minimize the impact of PM impairment in everyday life [8, 9].

PM is a complex ability that involves multiple processes and components. According to Ellis [10], there are five stages of PM: (1) formation and encoding of intention and action, (2) retention interval, (3) performance interval, (4) initiation and execution of intended action, and (5) evaluation of the outcomes. The first stage involves realizing that an action needs to be carried out in the future and encoding what the action is and when to execute it. In the second stage, the person stores the intended action and engages in one or more other activities. The action is initiated and executed in the third and fourth stages. During the last stage, the person records and evaluates the outcome of the intended action. Therefore, PM involves a combination of RM (to encode and recall the action), executive function (to plan and initiate the action at the correct time), and metacognitive function (to monitor performance and evaluate the outcome). These functions are commonly impaired after TBI; the prefrontal lobes which are vulnerable to trauma are implicated in executive and metacognitive functions and are also considered the neural basis of PM [11].

Three subtypes of PM have been identified in the scientific literature, according to task characteristics and the type of cue for recalling the intention [12]. These include: time-based PM activities when an action needs to be initiated at a particular time or window of time (e.g., in 10 min or before the close of business on Friday); event-based PM activities where an action is performed in response to an external cue (e.g., turn off the stove when the timer rings or ring a friend for their birthday when a reminder appears on your phone); and activity-based PM activities where an action is performed as part of or at the end of a sequence of actions or behaviors (e.g., put on your seatbelt after you get in the car or add sugar to your coffee after the milk). A study of the subtypes of PM using a computerized task showed that people with TBI performed significantly worse than controls on all subtypes [12]. In another study, patients with TBI had more difficulties compared to controls when the cognitive demand of the ongoing task was high, indicating their PM failures could be attributed to reduced processing capacity and problems allocating cognitive resources [4]. In previous research, significant others of patients with TBI reported that patients had significantly more instances of forgetting daily activities compared to non-injured controls [5]. In contrast, patients with TBI rated their own frequency of forgetting as similar to controls and reported fewer PM failures than their significant others, thus indicating a lack of self-awareness of their PM problems [5].

Like traditional RM rehabilitation, approaches to PM rehabilitation can be categorized as remedial or compensatory [13]. Remedial training approaches aim to restore or ameliorate the underlying impairment through repetitive practice of PM activities; for example, remembering to perform time- and event-based PM tasks embedded within a filler activity that may be pen-and-paper- or computer-based. In contrast, compensatory approaches focus on teaching the patient to use strategies to minimize the impact of PM impairment in everyday life; for example, training in diary or calendar use [13]. Case studies have found positive effects of remedial PM training [14, 15]; however, the training programs involved in these studies required substantial commitments of time and effort; for example, 4–6 h per week over several months. It is also not clear to what extent gains generalized beyond the ability to perform the simple, non-goal directed remedial activities used in training. Compensatory approaches to PM rehabilitation have been less investigated and overall the evidence is weak [16]. This is despite the common prescription of compensatory memory strategies in brain injury rehabilitation settings and the recommendation that training in external memory compensations should be standard practice [17]. Such strategies include the use of a diary, note-taking, routines, checklists, alarms, and paging systems, and are more relevant to everyday functioning, and may be more easily generalized to naturalistic settings compared to remedial activities. Furthermore, compensatory approaches to PM rehabilitation can assist patients with reduced cognitive capacity [18].

Trials with small TBI samples and case studies have described the use of electronic devices (e.g., palmtop computer, television-assisted prompting, Voice Organizer, and the NeuroPage paging system) as effective to compensate for PM deficits [19–24]. For an overview of these studies see the systematic review by de Joode et al. [25]. Fish et al. [26] evaluated the use of a “content-free” cue in the form of a text message, to remind 20 people with brain injury to monitor behavior, and found a greater improvement in performance on cued days compared to non-cued days. Ownsworth and McFarland [27] demonstrated that a metacognitive approach was more effective in training people with TBI to regularly use a memory notebook (*n* = 10) than a procedural learning approach (*n* = 10). Das Nair and Lincoln [28] compared the effectiveness of compensatory training, remedial training, and a self-help group in a group of 72 adults with memory problems (16 with TBI). They found that greater use of internal memory aids was reported post test by individuals in the two training groups compared to the self-help group, and that significantly better emotional status was reported by those in the compensation group.

In previous research, we developed an 8-week intervention program (consisting of two self-awareness training sessions and six compensatory training sessions) with the aim of rehabilitating the PM of three patients with TBI [8]. The promising findings of this pilot led to a larger-scale randomized controlled trial (RCT) of PM rehabilitation strategies [9], which demonstrated that the six-session compensatory rehabilitation program was more effective than a control intervention in improving PM in people with TBI, as measured by the Cambridge Prospective Memory Test (CAMPROMPT). The control intervention involved a remedial approach of repetitive practice of PM activities over six weekly sessions. The compensatory intervention involved the use of external strategies to both trigger the intention (prospective component) and support retrieval of content salient to the task (retrospective component) over the same time period. Event-based tasks (i.e., taking a cake out of the oven when a timer goes off) are easier than self-initiated or time-based tasks (i.e., remembering to take the cake out of the oven in 40 min) [12]. Therefore, this approach uses compensatory strategies that convert time-based tasks into event-based tasks which provide a cue to initiate performance of the action. Examples are use of an alarm, or linking the task to an event (e.g., taking medication at mealtimes). The significantly greater improvement in PM performance for the compensatory intervention group was attributed to the use of strategies during the PM task. However, these gains did not extend to significantly greater improvements in everyday PM performance or psychosocial function as anticipated. Further research is needed to specifically investigate the generalization of PM compensatory strategies to occupational performance in daily life [29].

One of the difficulties with generalization in TBI rehabilitation is the problem of impaired self-awareness. Estimates of the rates of impaired self-awareness in people with severe TBI are as high as 97% [30]. Impaired self-awareness refers to an inability to recognize limitations due to brain injury and to understand the functional implications of these [31]. It poses significant obstacles to rehabilitation engagement and outcomes in the TBI population and, therefore, is the target of rehabilitation efforts [32]. For example, individuals with poor self-awareness of their impairments post TBI do not see the need to apply strategies learnt in rehabilitation in everyday life to improve their performance. A fundamental principle of cognitive rehabilitation is that individuals need to recognize their impairments before being able to independently employ a strategy to compensate for these problems in daily living [33]. Therefore, to enhance the rehabilitation of PM, it is important that impairments of self-awareness are addressed to maximize engagement and use of strategies in everyday life.

Metacognitive skills training (MST) is a cognitive rehabilitation approach which facilitates the development of self-awareness in patients with TBI [33–35]. The objective of MST is to teach individuals how to self-monitor their performance, identify and self-correct errors, and generate strategies for future use. A metacognitive approach targets both on-line awareness (i.e., recognition and self-correction of errors in task performance) and intellectual awareness (i.e., self-knowledge of strengths and limitations). This approach incorporates elements of self-awareness training, such as psychoeducation and role modeling of strategy use, and also uses timely verbal, video, and experiential feedback and self-prediction and self-evaluation of performance in therapy activities and everyday life [35]. A MST approach has potential to enhance the outcomes of compensatory PM rehabilitation and lead to better generalization of strategy use beyond the therapy environment.

This paper outlines the protocol for a RCT comparing the efficacy of ﻿compensatory (COMP) and COMP-MST training for improving everyday PM performance, psychosocial function, strategy use, self-awareness, and level of care in a sample of adults with TBI.

### Study objectives and hypotheses

The primary aim of this RCT is to evaluate the effectiveness of a MST approach to compensatory PM training for improving everyday PM performance, maintaining PM gains, and improving level of psychosocial reintegration in community-dwelling adults with moderate to severe TBI. The secondary aim is to evaluate the effectiveness of a MST approach to compensatory PM training for improving and maintaining PM function on psychometric testing, strategy use, self-awareness, and level of care following TBI.

It is proposed that, with the addition of MST to the compensatory program, individuals with TBI will become aware of the extent of their PM failures, and the gains in PM performance will extend to everyday life as the participants will be able to generalize their strategy use to situations beyond the training setting. Therefore, it is hypothesized that:

1. COMP training will demonstrate significant improvement at post intervention and maintenance at 3-months post intervention on PM performance and level of psychosocial integration compared to no intervention (i.e., waitlist), and that compensatory PM rehabilitation combined with metacognitive skills training (COMP-MST) will be more effective than COMP training alone
2. Compared to participants in the waitlist condition, participants who receive COMP training will demonstrate significant improvement and maintenance of PM function on psychometric testing, strategy use, self-awareness, and level of support needs and, furthermore, that COMP-MST training will be more effective than COMP training alone

## Methods/design

### Trial design

**Fig. 1:**
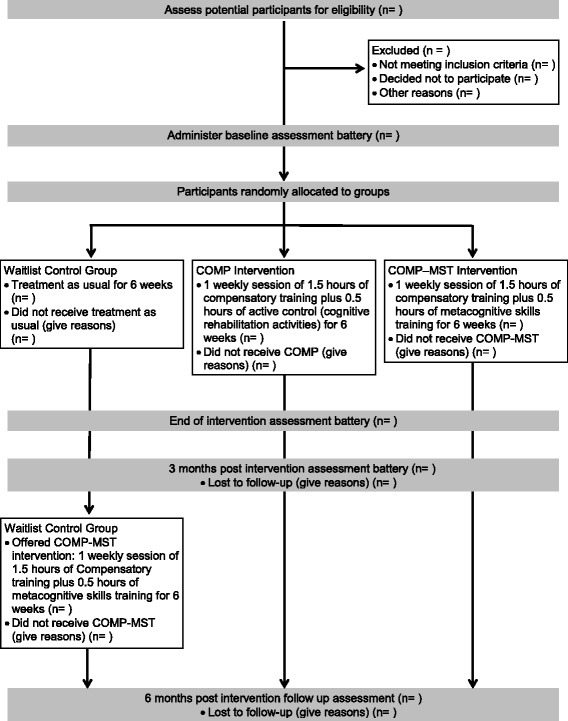
Trial design flowchart (*COMP* compensatory training, *COMP-MST* compensatory training plus metacognitive skills training)

**Fig. 2:**
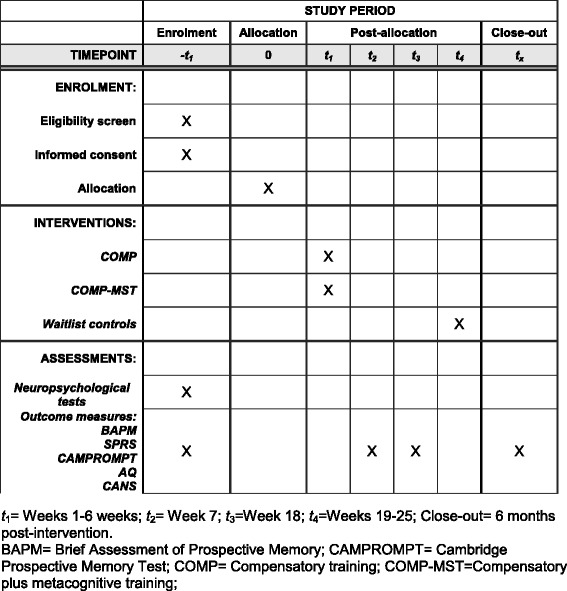
Standard Protocol Items: Recommendations for Interventional Trials (SPIRIT) diagram

The research design entails a RCT with three treatment groups: COMP-MST, COMP, and waitlist control. After completing baseline assessment measures (see “Measures” section), participants will be randomly allocated to the COMP-MST, COMP, or waitlist control group (see “Randomization” section). The COMP-MST and COMP groups will participate in a 6-week treatment intervention (see “Intervention procedures” section), and the waitlist control group will receive usual care for 6 weeks. At the end of the 6-week period, all participants will be reassessed on measures used at baseline by a blind assessor. Three months after conclusion of the intervention programs and waitlist control condition, all participants will receive a follow-up assessment to evaluate the maintenance of treatment effects. At this time, participants in the waitlist group will be offered the COMP-MST program. Six months after completing the intervention programs, all treatment groups will receive further follow-up assessment to determine maintenance of gains. The trial design flowchart is listed in Fig. 1 and the Standard Protocol Items: Recommendations for Interventional Trials (SPIRIT) diagram in Fig. 2.

### Participants

#### Recruitment

Recruitment of 90 participants with moderate to severe TBI and their significant others will be carried out over a 4-year period. Potential participants will be identified by treating therapists or case managers providing rehabilitation to people with TBI in a specialist brain injury rehabilitation unit at a large metropolitan hospital in Queensland, and a Queensland statewide brain injury outreach service. A treating therapist or case manager will provide potential participants with a brief summary of the study. If the individual is interested in taking part, they will be asked to provide verbal consent to be contacted by the project manager. Flyers advertising the project will also be provided to private brain injury rehabilitation practices in Brisbane, Australia and interested participants will be invited to contact the project manager. The project manager will then explain the study and provide participants with a Participant Information Sheet and a Consent Form (see Additional file 1).

#### Eligibility criteria

Participants will be eligible for the study if they are aged 18–65 years; have a significant other who is available to participate in the study; have a diagnosis of moderate or severe TBI (as determined by Glasgow Coma Scale (GCS) score and/or duration of post-traumatic amnesia (PTA)); score within the impaired range on baseline assessment of PM performance or if PM problems are reported by the participant or their significant other on the Brief Assessment of Prospective Memory (BAPM); are at least 1 month post discharge from hospital; have had no prior brain injury or hypoxic injury; have adequate receptive and expressive English communication skills; are ambulant or independently mobile in a manual or electric wheelchair; and are able to attend the hospital for the 6-week intervention program. Suitability for the study will be confirmed at the preintervention assessment.

Participants will be excluded from the study if they are unable to provide informed consent; have not emerged from PTA; are confused or disoriented; have communication difficulties limiting their comprehension of written or spoken language; and/or who are assessed by their treating occupational therapist as having impairment of cognitive function at a basic level.

### Ethics, consent, and permissions

The ethical aspects of this research project have been approved by the Human Research Ethics Committees, Queensland (HREC) of Metro South (HREC/15/QPAH/90 – SSA/15/QPAH/92), the University of Queensland (2015000644), and Griffith University (PSY/93/14/HREC), Queensland, Australia. The trial is registered with the Australian and New Zealand Clinical Trials Registry (ACTRN12615000996561). The trial is being monitored by the research monitoring officer, Centre for Health Research, Princess Alexandra Hospital. This study protocol conforms to the SPIRIT reporting guidelines. See the SPIRIT Checklist in Additional file 2.

Informed consent will be obtained from each participant prior to commencement in the study. See the Participant Information Sheet and Consent Form in Additional file 1. The blind assessor will conduct preintervention assessments with each participant in the clinic, which will involve completing a series of questionnaires and neuropsychological tests. The participant’s significant other will also provide informed consent and complete a set of questionnaires (see “Measures” section).

Personal information about potential and enrolled participants will remain confidential and data will be deidentified using participant numbers. A dedicated project manager will oversee data collection, organization, storage, and security. Only the project researchers and the research monitoring officer will have access to the data. At the conclusion of the study, data will be stored in accordance with the National Health and Medical Research Council retention of study data policy. Participants will be provided with a summary of their results approximately 6 months after their final assessment. It is anticipated that the results of this research will be published and/or presented in a variety of forums. In any publications and/or presentations, information will be provided in such a way that participants cannot be identified.

### Procedure

#### Randomization

Participants’ impairment of PM function as assessed on the CAMPROMPT (“impaired/borderline” or “less impairment”) will be used to determine their stratification subgroup for random allocation to the three groups. This stratified approach is designed to ensure a relatively equal proportion of individuals in each group with severe PM impairment, as determined using cutoffs in the CAMPROMPT manual [36]. The random assignment will be conducted independent of the project staff involved in the interventions and the blind assessor. For each subgroup (i.e., severe or less severe), participants will be randomly allocated to COMP, COMP-MST, or waitlist control groups using sequentially numbered and sealed opaque envelopes. The envelopes will contain group allocation on a written insert, based on a predetermined, random, computer-generated sequence.

#### Intervention procedures

Manualized treatment protocols have been developed for the COMP and COMP-MST training programs based on previous research conducted by the research team [9]. The COMP treatment manual has been amended for use in the current study to incorporate electronic devices used as compensatory aides (i.e., mobile phones). The MST component of the COMP-MST treatment manual has been developed by the research team [34]. The techniques of the COMP and COMP-MST training procedures are shown in Table 1.

**Table 1:** Overview of COMP and COMP-MST training techniques

Compensatory strategy training (completed in COMP and COMP-MST)	
Description	Aim
Prospective memory education	To develop participants’ understanding of what PM is and the impact of TBI on PM ability
Use of a memory aid E.g., diary or organizational device	To assist participants in identifying and learning to use a portable compensatory aid that meets their individual needs and preferences
Time management and environmental organization techniques	To maximize organization and time management within participants’ existing routines and home environments
Writing reminders, appointments, note-taking	To demonstrate basic note-taking skills for recording reminders
Family/friend training	To involve significant others in participants’ memory aid training in order to increase others’ understanding of PM strategies and reinforce the use of memory aids outside of the training environment
Strategy generation practice E.g., video scenarios of everyday memory failures	To encourage self-generation of a range of suitable strategies for use within the context of “real-life” PM failures that impact on independent living, community integration, and social relationships
Future memory aid agreement	To reinforce use of the memory aid and note-taking skills, and devise a plan for maintenance of strategy use in the future
Cognitive activity (completed in COMP only)	
Description	Aim
Cognitive activity E.g., workbook exercises, attention tasks	To provide an active control or filler task for metacognitive skills training, i.e., a similar amount of therapy time allocated to tasks unrelated to PM or self-awareness
Metacognitive skills training (completed in COMP-MST only)	
Description	Aim
Role modeling E.g., watching a video of someone else discussing their PM difficulties and demonstrating ways they overcame their PM failure	To allow participants to identify similarities between the PM problems being modeled and their own experience, to increase self-awareness of personal challenges and potential usefulness of strategies
Timely feedback E.g., verbal, video, experiential and written feedback	To provide an opportunity for participants to gain insight into their PM performance, self-reflect on their performance, and generate strategies for future use
Self-reflection activities E.g., self-prediction of PM performance prior to performance and self-evaluation following performance; journal of PM failures and strategy use in everyday life; discussion of impact of TBI	To encourage participants to self-monitor their performance, gain insight into how to self-correct errors, and practice generating strategies for future use

Registered occupational therapists who are experienced in working with people with brain injury will deliver the intervention program by one-to-one consultation in an outpatient clinic setting. Treatment fidelity will be monitored using a checklist based on Borelli’s framework [37]. Sessions will be audiotaped to enable therapists’ adherence to the treatment protocol to be examined for a random sample (20%) of sessions by experts who are independent of the study. At the end of the intervention, the effectiveness of blinding will be checked by asking the assessor to state if they became aware of which group the participant belongs to. Throughout the study, participant compliance will be monitored by recording the number of sessions that each participant attends. Strategies to improve participation include the provision of financial support for transportation to therapy sessions, and extending the intervention period from 6 weeks to 8 weeks in the case of illness or other absence. Participants can continue to receive other rehabilitation services which are not focused on PM rehabilitation during the intervention period and these will be documented.

### Measures

The primary outcome measures assess everyday PM performance and level of psychosocial reintegration. Secondary outcome measures assess PM performance and strategy use, global self-awareness, self-awareness of PM failure, and level of support needs. Primary and secondary outcome measures will be administered at pre and post intervention, and at 3-month and 6-month follow-ups.

#### Primary outcomes

The Brief Assessment of Prospective Memory (BAPM; [38]) is a 16-item self-report scale which measures frequency of PM failure in everyday life over the past month. The BAPM consists of two subscales: basic activities of daily living (eight items) and instrumental activities of daily living (eight items). Subscale scores range from 0 to 5, with higher scores reflecting more frequent PM failure. Internal consistency and test-retest reliability have been reported [37]. Significant other ratings will be used in this study, rather than self-ratings, in order to overcome confounding scores due to the development of self-awareness in TBI participants over the course of the intervention.

The Sydney Psychosocial Reintegration Scale version 2 (SPRS-2; [39, 40]) is a 12-item questionnaire which assesses an individual’s level of psychosocial reintegration following TBI. The SPRS-2 contains two forms which reflect different comparison standards: Form A measures change since injury and Form B measures current status. Each form uses the same 12 items which measure three domains of functioning including occupational activities, independent living skills, and interpersonal relationships. Each domain contains four items, which are rated on a 5-point scale ranging from “no change” to “extreme change” (Form A) and “very good” to “extremely poor” (Form B). Scores range from 0–48, with higher scores indicative of better psychosocial functioning. The SPRS-2 (Form B) will be completed by participants’ significant others. Sound psychometric properties have been reported for the SPRS-2 [39].

#### Secondary outcomes

The Cambridge Prospective Memory Test (CAMPROMPT; [36]) is a psychometric test of PM function. The CAMPROMPT consists of a total of six PM tasks, three cued by time and three cued by events. Participants are allowed to spontaneously use strategies, such as taking notes, to help them remember during tasks. Total scores are out of 36 (time-based and event-based subscale scores are each out of 18), with higher scores representing better PM performance. The validity and reliability of the CAMPROMPT has been documented in a number of studies [36, 41]. In addition to total CAMPROMPT scores, participants’ spontaneous use of note-taking as a PM strategy during the CAMPROMPT will be measured as a dichotomous variable.

The Awareness Questionnaire (AQ; [42]) is a 17-item scale that assesses self-awareness of brain injury-related impairments across sensory, physical, cognitive, and behavioral domains. Ratings on the AQ compare participants’ post-injury abilities on each item to their preinjury functioning (1 = much worse, 5 = much better). The AQ has two versions, a self-report and a significant-other report which may be completed by a family member, friend, or therapist. Positive discrepancy scores (self-report minus significant-other report) indicate impaired self-awareness. The AQ has been found to have sound psychometric properties [43] and has demonstrated sensitivity to change in the context of awareness interventions [35].

Self-awareness specifically for PM impairment will be measured using the BAPM by calculating the discrepancy between participant self-ratings and significant other ratings of the frequency of PM failure in everyday life.

The Care and Needs Scale (CANS; [44]) is a clinician rating scale, designed to measure the types and level of support needs of a person with TBI. The types of support needs are assessed using a 24-item checklist, hierarchically grouped into categories reflecting the intensity of support required (such as support with special needs, basic activities of daily living, instrumental activities of daily living, and psychosocial supports). The information on types of support needs required by the individual is then used to evaluate the level of support needs, as indexed by the length of time that a person can be left alone. The CANS rating ranges from 0 (is completely independent) to 7 (cannot be left alone). Reliability of the CANS has been investigated by Soo et al. [45]. The CANS will be completed in conjunction with the participant’s referring occupational therapist.

### Data collection and management

All measures will be completed at the four time points by an assessor who is blind to group allocation. At the preintervention assessment, the assessor will also administer a brief neuropsychological battery consisting of tests of attention (e.g., Digit Span, Trail Making Test A), memory (e.g., Hopkins Verbal Learning Test), and executive function (e.g., Controlled Oral Word Association Test, Trials B, Hayling Sentence Completion Test) in order to provide data on the level of cognitive impairment of the sample. Data on demographic and diagnostic variables (GCS score, duration of PTA, length of hospitalization, and cause of injury) will be retrieved from the participant’s medical records. Significant others will complete the SPRS-2 and BAPM at the clinic if they attend the assessment sessions with the participant with TBI. If they do not attend they will complete the measures at home, with telephone assistance from the assessor if required, and return the questionnaires by post.

Participant retention will be promoted via transport assistance where required and follow-up telephone contact from the project manager. The data management procedure includes cross checking of data using computed and manually scored totals and range checks for data values. Any adverse events will be reported to the research monitoring officer of the Human Ethics Research Committee.

### Sample size

To determine the required sample size, a power calculation was conducted using the primary outcome variable with the most conservative effect size from our previous RCT (i.e., the change score between pre-and post-SPRS-2 scores) [9]. Comparison between the group that received compensatory plus self-awareness training and the group that received compensatory training alone produced an effect size of 0.70 (Cohen’s *d*). Using G*Power [46] and setting the effect size to 0.70, alpha level to 0.05, and power to 0.80 it was calculated that a sample size of *n* = 26 for each group is required. The sample size has been inflated to *n* = 30 per group to allow for an estimated dropout rate of approximately 10% based on dropout rates from another RCT currently being conducted within the same setting [47]. Therefore, it is calculated that a total sample size of *n* = 90 will be required for the study to be adequately powered to detect significant differences between groups.

### Statistical analysis

Between-group differences will be calculated, accounting for baseline scores, for all three groups on all outcomes. To address the primary aim, unstructured linear mixed regression will be used to test for group differences on the primary outcomes, with group allocation (COMP-MST, COMP, waitlist control) as the between-subject factor, and time (baseline, post intervention, 3-month follow-up) as the within-subject or repeated variable. Any demographic or neuropsychological variables that are significantly associated with the primary outcome and unevenly distributed between the groups will be included as covariates. The same approach will be used with secondary outcome variables to address the secondary aim. Missing data will be handled using an intention-to-treat analysis (i.e., most recent available data will be imputed). Using planned comparisons, data from the 6-month follow-up will be compared with post-intervention and/or 3-month follow-up results to evaluate whether or not any significant gains on variables have been maintained over time.

### Interim analysis and stopping rules

We do not have a plan for an interim analysis as we do not expect a situation that would lead us to stop the study. However, in the event of an extreme situation, we will temporarily cease data collection to assess the implications on the study design.

## Discussion

Impairment of PM is common following TBI and may have serious repercussions for an individual’s independent living, social engagement, and employability [48]. Whilst research shows that compensatory approaches to PM rehabilitation minimize the impact of PM impairments in adults with TBI [8, 9], poor self-awareness after TBI poses a major barrier to the generalization of compensatory strategies in daily life [32]. By comparing the efficacy of COMP and COMP-MST, this project will determine if integrating MST into compensatory approaches to PM rehabilitation improves everyday PM performance, strategy use, and self-awareness after TBI. The findings are expected to have important theoretical and practical implications for treatment approaches that can potentially be used to enhance individuals’ capacity to generalize compensatory training strategies to daily life, which in turn will increase independent living and reduce the personal and social burden of TBI in the community.

The main methodological challenge in this study relates to avoiding therapist bias, as the same occupational therapist will deliver both intervention programs to participants. The use of manualized procedures, close supervision, fidelity checks to ensure adherence to protocol, and the therapists being blinded to hypotheses will reduce potential therapist bias. Furthermore, using the same therapist provides consistency across interventions (e.g., consistent level of expertise, rapport, other therapist factors).

### Trial status

Patient recruitment was ongoing at the time of manuscript submission. Recruitment for the study commenced in July 2015.

## Additional files

Additional file 1: Participant Information Sheet and Consent Form. (DOC 112 kb)
Additional file 2: SPIRIT Checklist. (DOC 138 kb)

## Abbreviations

AQ: Awareness Questionnaire
BAPM: Brief Assessment of Prospective Memory
CAMPROMPT: Cambridge Prospective Memory Test
CANS: Care and Needs Scale
COMP: Compensatory approaches to prospective memory rehabilitation
COMP-MST: Compensatory approaches to prospective memory rehabilitation plus metacognitive skills training
GCS: Glasgow Coma Scale
HREC: Human Research Ethics Committees
MST: Metacognitive skills training
PM: Prospective memory
PTA: Post-traumatic amnesia
RCT: Randomized controlled trial
RM: Retrospective memory
SPRS-2: Sydney Psychosocial Reintegration Scale version 2
TBI: Traumatic brain injury
