# Supplementary material for: The efficacy of prospective memory rehabilitation plus metacognitive skills training for adults with traumatic brain injury: study protocol for a randomized controlled trial
Source: Trials. 2017 Jan 5;18:3. doi: 10.1186/s13063-016-1758-6 (PMC5217156; doi:10.1186/s13063-016-1758-6)
Supplement: Additional file 1: — Participant Information Sheet and Consent Form. (DOC 112 kb) [file 13063_2016_1758_MOESM1_ESM.doc]

**Participant Information Sheet/Consent Form**

**Health/Social Science Research**

| **Title** | Efficacy of prospective memory rehabilitation plus metacognitive skills training for adults with traumatic brain injury: a randomized controlled trial |
| --- | --- |
| **Short Title** | Prospective memory and metacognitive skills training after traumatic brain injury |
| **Protocol Number** | HREC/15/QPAH/090 |
|  |  |
| **Coordinating Principal Investigator and Associated Investigators** | Assoc Professor Jennifer Fleming, Conjoint Associate Professor in Occupational Therapy, Princess Alexandra Hospital and The University of Queensland  Prof David Shum, Dean (Research), School of Applied Psychology and Griffith Health Institute, Griffith University  Assoc Professor Tamara Ownsworth, School of Applied Psychology and Griffith Health Institute, Griffith University  Dr Emmah Doig, Postdoctoral Research Fellow, School of Health and Rehabilitation Sciences, The University of Queensland  Ms Janelle Griffin, Team Leader, Brain Injury Rehabilitation, Occupational Therapy Department, Princess Alexandra Hospital  Assoc Professor Melissa Kendall, Senior Research Officer, Acquired Brain Injury Outreach Service |
| **Location** | Princess Alexandra Hospital  Occupational Therapy Department |

**Part 1 What does my participation involve?**

**1. Introduction**

You are invited to take part in this research project which is called “Efficacy of prospective memory rehabilitation plus metacognitive skills training for adults with traumatic brain injury: a randomized controlled trial”. You have been invited because you have had a traumatic brain injury (TBI) and are having difficulty with your memory.

This Participant Information/Consent Form tells you about the research project. It explains the processes involved with taking part. Knowing what is involved will help you decide if you want to take part in the research.

Please read this information carefully. Ask questions about anything that you don’t understand or want to know more about. Before deciding whether or not to take part, you might want to talk about it with a relative, friend or local health worker.

Participation in this research is voluntary. If you don’t wish to take part, you don’t have to.

If you decide you want to take part in the research project, you will be asked to sign the consent section. By signing it you are telling us that you:

• Understand what you have read

• Consent to take part and being involved in the research project

• Consent to the use of your personal and health information as described.

You will be given a copy of this Participant Information and Consent Form to keep.

**2 What is the purpose of this research?**

The project aims to look at the effectiveness of a rehabilitation program for improving prospective memory function in everyday activities. Prospective memory refers to remembering to carry out things you intend to do in the future, for example remembering to attend an appointment, or remembering to take medication.

Previous research has shown that survivors of TBI often have memory difficulties which may impact on their independent living, social participation and employment. This project will consider whether rehabilitation to develop self-awareness and strategy use will improve everyday function and if these improvements are maintained (3 months and 6 months) after the rehabilitation has finished.

This research has been initiated by the researchers, Dr Jenny Fleming and Dr Emmah Doig (University of Queensland), Prof David Shum and Dr Tamara Ownsworth (Griffith University), Ms Janelle Griffin (Princess Alexandra Hospital) and Dr Melissa Kendall (Acquired Brain Injury Outreach Service).

This research has been funded by the National Health and Medical Research Council.

**3 What does participation in this research involve?**

If you agree to participate in this research, a consent form will be signed prior to any study assessments being performed.

⮞ Initial steps:

- Screening for eligibility will be completed.

⮞ Procedures:

- If you consent to participate in the study, you will be randomly assigned to either one of two intervention groups or the waitlist group. Participants in the intervention groups will receive a weekly 2-hour rehabilitation sessions for 6 weeks, while the waitlist group will continue to receive their usual rehabilitation. After 3 months, those in the waitlist group will be offered the chance to participate in the intervention.
- All rehabilitation sessions will take place at the Centre for Functioning and Health Research in Buranda shopping village. Each session will go for approximately 2 hours.
- Assessments: You will receive a cognitive assessment (e.g. memory and attention tests) conducted by a psychologist before commencing the project. Other assessments will be completed four times during the project. They involve a memory assessment and four questionnaires about your memory functioning and functional abilities. Assessments will take place before commencing rehabilitation, at completion (6 weeks) and at 3 months and 6 months. Participants in the waitlist group will complete the questionnaires on one additional occasion 6 weeks after commencing the project. Assessments will take approximately 30 minutes.
- Significant Other participation: You will be asked to provide details of a family member or friend who has daily contact with you who will be asked to participate in this study. Their participation will include completing questionnaires about your memory and functional abilities before you commence rehabilitation, at 6 weeks and at 3 months and 6 months follow up. This is required in order to get an accurate picture of how you function in your day-to-day life from someone who knows you very well.
- A review of your medical chart will be undertaken by the researcher in order to obtain information about your injury, and demographic factors such as your age.
- It is anticipated that your involvement in the project will last up to 12 months. The entire research project is expected to be completed within 4 years.
- Reimbursement and costs: There are no costs associated with participating in this research project, nor will you be paid. However, you can be reimbursed for any reasonable travel or parking expenses associated with the research project visit up to $20/session.
- The research will be monitored by the Metro South Human Research Ethics Committee in accordance with NHMRC [National Statement on Ethical Conduct in Human Research](http://www.nhmrc.gov.au/publications/synopses/e72syn.htm) (2007).

This research project has been designed to make sure the researchers interpret the results in a fair and appropriate way and avoids the researchers and participants jumping to conclusions.

**4 Other relevant information about the research project**

There will be 90 participants in this study divided in to three groups – two Intervention Groups and the Waitlist Group. All participants will receive rehabilitation (in one of the two intervention groups) and participate in assessments to review progress.

Participation of a significant other is needed to be a part of this study. If your significant other decides not to participate, or withdraws consent during the study, you can nominate another person who knows you well, or continue without a significant other.

**5 Do I have to take part in this research project?**

Participation in any research project is voluntary. If you do not wish to take part, you do not have to. If you decide to take part and later change your mind, you are free to withdraw from the project at any stage.

If you do decide to take part, you will be given this Participant Information and Consent Form to sign and you will be given a copy to keep.

Your decision whether to take part or not to take part, or to take part and then withdraw, will not affect your routine care, your relationship with professional staff or your relationship with the Princess Alexandra Hospital Brain Injury Rehabilitation Unit or Acquired Brain Injury Outreach Service.

**6 What are the possible benefits of taking part?**

We cannot guarantee or promise that you will receive any benefits from this research; however, benefits may include the opportunity to:

- participate in weekly rehabilitation with the potential to learn strategies to aid your memory.
- give you feedback regarding your participation and use of memory strategies.

The project may also lead to a better understanding of the benefits or otherwise of this form of rehabilitation for adults following TBI, and help others in the future.

**7. What are the possible risks and disadvantages of taking part?**

If you are assigned to the waitlist group you will have a 3-month wait before you can commence the intervention program.

**8. What if I withdraw from this research project?**

If you do consent to participate, you may withdraw at any time. If you decide to withdraw from the project, please notify a member of the research team before you withdraw. A member of the research team will inform you if there are any special requirements linked to withdrawing. If you do withdraw, you will be asked to complete and sign a ‘Withdrawal of Consent’ form; this will be provided to you by the research team.

If you decide to leave the research project, the researchers will not collect additional personal information from you, although personal information already collected will be retained to ensure that the results of the research project can be measured properly and to comply with law. You should be aware that data collected up to the time you withdraw will form part of the research project results. If you do not want your data to be included, you must tell the researchers when you withdraw from the research project.

**9. Could this research project be stopped unexpectedly?**

It is not anticipated that this research project will be stopped unexpectedly.

**10 What happens when the research project ends?**

At the conclusion of the project, participants will be provided with a summary of the results. This will be mailed to you once data analysis has been completed approximately 6 months after your final assessment.

**Part 2 How is the research project being conducted?**

**11 What will happen to information about me?**

By signing the consent form you consent to the research team collecting and using personal information about you for the research project. Any information obtained in connection with this research project that can identify you will remain confidential. Data collected will be coded and not linked to you personally. Data will be stored securely on location in the Centre for Functioning and Health Research. Only the project researchers listed on this consent form will have direct access to all documents. At the conclusion of the study, data will be stored for a period of 7 years, in accordance the Princess Alexandra Hospital retention of study data policy.

Your information will only be used for the purpose of this research project and it will only be disclosed with your permission, except as required by law. The personal information that the research team collect and use is information from your medical record, baseline assessments questionnaires and rehabilitation sessions.

Information about you may be obtained from your health records held at the hospital for the purpose of this research. By signing the consent form you agree to the research team accessing health records if they are relevant to your participation in this research project.

It is anticipated that the results of this research project will be published and/or presented in a variety of forums. In any publication and/or presentation, information will be provided in such a way that you cannot be identified.

In accordance with relevant Australian and/or Queensland privacy and other relevant laws, you have the right to request access to the information about you that is collected and stored by the research team. You also have the right to request that any information with which you disagree be corrected. Please inform the research team member named at the end of this document if you would like to access your information.

**12 Complaints and compensation**

If you suffer any distress or injury as a result of this research project, you should contact the research team as soon as possible. You will be assisted with arranging appropriate treatment and support. Please contact the Metro South Human Resource Ethics Committee to report any complaints on phone 07 3443 8049 or email PAH_Ethics_Research@health.qld.gov.au.

**13 Who is organising and funding the research?**

This research project is being conducted by a research team at the Princess Alexandra Hospital funded by the National Health and Medical Research Council.

No member of the research team will receive a personal financial benefit from your involvement in this research project (other than their ordinary wages).

**14 Who has reviewed the research project?**

All research in Australia involving humans is reviewed by an independent group of people called a Human Research Ethics Committee (HREC).The ethical aspects of this research project have been approved by the HREC of Metro South Hospital and Health Services District.

This project will be carried out according to the *National Statement on Ethical Conduct in Human Research (2007)*. This statement has been developed to protect the interests of people who agree to participate in human research studies.

**15 Further information and who to contact**

The person you may need to contact will depend on the nature of your query. If you want any further information concerning this project or if you have any problems which may be related to your involvement in the project, you can contact the following people:

**Research contact person**

| Name | Jennifer Fleming |
| --- | --- |
| Position | Conjoint Associate Professor |
| Telephone | (07) 3008 6955 |
| Email | j.fleming@uq.edu.au |

If you have any complaints about any aspect of the project, the way it is being conducted or any questions about being a research participant in general, then you may contact:

| Reviewing HREC name | Metro South Hospital and Health Service |
| --- | --- |
| HREC Executive Officer | Ms Rebecca Lacey |
| Telephone | 07 3443 8049 |
| Email | PAH_Ethics_Research@health.qld.gov.au |

**Reviewing HREC approving this research** **and HREC Executive Officer details**

**Consent Form**

| **Title** | Efficacy of prospective memory rehabilitation plus metacognitive skills training for adults with traumatic brain injury: a randomised controlled trial |
| --- | --- |
| **Short Title** | Prospective memory and metacognitive skills training after traumatic brain injury |
| **Protocol Number** | HREC/15/QPAH/090 |
|  |  |
| **Coordinating Principal Investigator and Associated Investigators** | Assoc Professor Jennifer Fleming, Conjoint Associate Professor in Occupational Therapy, Princess Alexandra Hospital and The University of Queensland  Prof David Shum, Dean (Research), School of Applied Psychology and Griffith Health Institute, Griffith University  Assoc Professor Tamara Ownsworth, School of Applied Psychology and Griffith Health Institute, Griffith University  Dr Emmah Doig, Postdoctoral Research Fellow, School of Health and Rehabilitation Sciences, The University of Queensland  Ms Janelle Griffin, Team Leader, Brain Injury Rehabilitation, Occupational Therapy Department, Princess Alexandra Hospital  Assoc Professor Melissa Kendall, Senior Research Officer, Acquired Brain Injury Outreach Service |
| **Location** | Princess Alexandra Hospital |

**Declaration by Participant**

I have read the Participant Information Sheet or someone has read it to me in a language that I understand.

I understand the purposes, procedures and risks of the research described in the project.

I have had an opportunity to ask questions and I am satisfied with the answers I have received.

I freely agree to participate in this research project as described and understand that I am free to withdraw at any time during the project without affecting my future care.

I understand that I will be given a signed copy of this document to keep.

I consent for the researchers to access my medical records to retrieve information about driving assessments, injury details and demographic information.

|  | | | | | | | |
| --- | --- | --- | --- | --- | --- | --- | --- |
|  | Name of Participant (please print) print) | |  | |  |  |  |
|  | | | | | | | |
|  | Signature |  | | Date | |  |  |
|  | | | | | | | |

**Declaration by Researcher^†^**

I have given a verbal explanation of the research project, its procedures and risks and I believe that the participant has understood that explanation.

|  | | | | | | |
| --- | --- | --- | --- | --- | --- | --- |
|  | Name of Researcher^†^ (please print) | |  | | |  |
|  | | | | | |  |
|  | Signature |  | | Date |  |  |
|  | | | | | | |

^†^ An appropriately qualified member of the research team must provide the explanation of, and information concerning, the research project.

Note: All parties signing the consent section must date their own signature.

**Form for Withdrawal of Participation**

| **Title** | Efficacy of prospective memory rehabilitation plus metacognitive skills training for adults with traumatic brain injury: a randomised controlled trial |
| --- | --- |
| **Short Title** | Prospective memory and metacognitive skills training after traumatic brain injury |
| **Protocol Number** | HREC/15/QPAH/090 |
|  |  |
| **Coordinating Principal Investigator and Associated Investigators** | Assoc Professor Jennifer Fleming, Conjoint Associate Professor in Occupational Therapy, Princess Alexandra Hospital and The University of Queensland  Prof David Shum, Dean (Research), School of Applied Psychology and Griffith Health Institute, Griffith University  Assoc Professor Tamara Ownsworth, School of Applied Psychology and Griffith Health Institute, Griffith University  Dr Emmah Doig, Postdoctoral Research Fellow, School of Health and Rehabilitation Sciences, The University of Queensland  Ms Janelle Griffin, Team Leader, Brain Injury Rehabilitation, Occupational Therapy Department, Princess Alexandra Hospital  Assoc Professor Melissa Kendall, Senior Research Officer, Acquired Brain Injury Outreach Service |
| **Location** | Princess Alexandra Hospital |

**Declaration by Participant**

I wish to withdraw from participation in the above research project and understand that such withdrawal will not affect my routine care, or my relationships with the researchers or Princess Alexandra Hospital.

|  | | | | | | | |
| --- | --- | --- | --- | --- | --- | --- | --- |
|  | Name of Participant (please print) | |  | |  |  |  |
|  | | | | | | | |
|  | Signature |  | | Date | |  |  |
|  | | | | | | | |

In the event that the participant’s decision to withdraw is communicated verbally, the Senior Researcher must provide a description of the circumstances below.

|  |
| --- |

**Declaration by Researcher^†^**

I have given a verbal explanation of the implications of withdrawal from the research project and I believe that the participant has understood that explanation.

|  | | | | | | |
| --- | --- | --- | --- | --- | --- | --- |
|  | Name of Researcher (please print) | |  | | |  |
|  | | | | | |  |
|  | Signature |  | | Date |  |  |
|  | | | | | | |

^†^ An appropriately qualified member of the research team must provide information concerning withdrawal from the research project.

Note: All parties signing the consent section must date their own signature.
